# Androgen-induced miR-135a acts as a tumor suppressor through downregulating RBAK and MMP11, and mediates resistance to androgen deprivation therapy

**DOI:** 10.18632/oncotarget.9992

**Published:** 2016-06-14

**Authors:** Xuechao Wan, Honglei Pu, Wenhua Huang, Shu Yang, Yalong Zhang, Zhe Kong, Zhuoran Yang, Peiqing Zhao, Ao Li, Tao Li, Yao Li

**Affiliations:** ^1^ State Key Laboratory of Genetic Engineering, Shanghai Engineering Research Center of Industrial Microorganisms, School of Life Science, Fudan University, Shanghai, 200433, PR China; ^2^ Key Laboratory of Reproduction Regulation of NPFPC, Fudan University, Shanghai, 200433, PR China; ^3^ Center of Translational Medicine, Central Hospital of Zibo, Zibo, Shangdong, 255036, PR China

**Keywords:** miR-135a, RBAK, MMP11, PI3K/AKT, androgen receptor

## Abstract

The main challenge in the treatment of prostate cancer (PCa) is that the majority of patients inevitably develop resistance to androgen deprivation. However, the mechanisms involved in hormone independent behavior of PCa remain unclear. In the present study, we identified androgen-induced miR-135a as a direct target of AR. Functional studies revealed that overexpression of miR-135a could significantly decrease cell proliferation and migration, and induce cell cycle arrest and apoptosis in PCa. We identified RBAK and MMP11 as direct targets of miR-135a in PCa by integrating bioinformatics analysis and experimental assays. Mechanistically, miR-135a repressed PCa migration through downregulating MMP11 and induced PCa cell cycle arrest and apoptosis by suppressing RBAK. Consistently, inverse correlations were also observed between the expression of miR-135a and RBAK or MMP11 in PCa samples. In addition, low miR-135a and high RBAK and MMP11 expression were positively correlated with PCa progression. Also, PI3K/AKT pathway was confirmed to be an upstream regulation signaling of miR-135a in androgen-independent cell lines. Accordingly, we reported a resistance mechanism to androgen deprivation therapy (ADT) mediated by miR-135a which might be downregulated by androgen depletion and/or PI3K/AKT hyperactivation, in castration-resistant prostate cancer (CRPC), thus promoting tumor progression. Taken together, miR-135a may represent a new diagnostic and therapeutic biomarker for castration-resistant PCa.

## INTRODUCTION

Prostate cancer (PCa) is the second leading cause of cancer-related deaths in males with 27,540 attributed cases in the United States in 2015 [1]. The androgen receptor (AR), a member of the large family of nuclear receptors (NRs), plays a critical role in prostate carcinogenesis through the regulation of transcriptional networks, genomic stability, and gene fusions [2]. Androgen deprivation therapy (ADT), a standard treatment to disrupt AR signaling pathway, is the main treatment for the early-stage localized disease (androgen-dependent prostate cancer, ADPC) [2, 3]. However, PCa inevitably turns to be resistant to ADT after 18 to 24 months treatment with poor prognosis and high metastatic potential [3–5]. Hence, there is an urgent need for identifying new molecules and signals linked to hormone independent behavior of PCa.

MicroRNAs (miRNAs) are a class of well-conserved small noncoding RNAs (20–22 nucleotides long) [6], which regulate gene expression through binding to the 3′-untranslated region (3′-UTR) region of target transcripts [7] and promoting mRNA degradation or translational repression [8]. Recently, emerging evidence suggests that altered expression of miRNAs is an important mechanism of cancer pathogenesis. In prostate cancer, a few miRNAs including miR-125b [9], miR-21 [10], miR-32 [11] and miR-449 [12] were identified as androgen-responsive transcripts. In previous miRNA microarray analysis, we investigated an AR-microRNA-mRNA network[13] that regulated PCa cell viability, including miR-19a, miR-27a and miR-133b [14]. Notably, part of these miRNAs had shown association with transformation to androgen-resistant PCa [9–11]. Meanwhile, we also verified miR-135a as a candidate microRNA that was found to be responsive to androgen induction and a potential target of AR. Coincidently, Kroiss and his colleagues also identified miR-135a as an androgen-upregulated miRNA [15].

MiRNAs may function as either oncogenes or tumor suppressors in biologic processes of different cancer cells. MiR-135a was previously reported as an oncogene in breast cancer [16] and bladder cancer [17], or a putative tumor suppressor in human gallbladder cancer [18], epithelial ovarian cancer [19] and gastric cancer [20]. Additionally, Kroiss had demonstrated that miR-135a decreased prostate cancer cell migration and invasion through downregulating ROCK1 and ROCK2 [15]. However, the complex biologic processes mediated by miR-135a that inhibited tumor proliferation were not well described. Understanding the detailed molecular mechanisms underlying the effects of miR-135a on the progression of PCa is essential in developing miR-135a as a diagnostic and therapeutic target.

In the present study, we investigated the potential effect of miR-135a on hormone independent behavior of PCa. We demonstrated that miR-135a was an androgen-induced tumor suppressor in prostate cancer. Our data highlighted a functional role of miR-135a that not only repressed PCa migration and invasion but also induced PCa cells cycle arrest and apoptosis by directly targeting the 3′UTR of MMP11 and RBAK mRNA. Furthermore, we explored the potential signaling pathways regulating miR-135a expression in androgen-independent PCa cells and we found that PI3K/AKT signaling significantly reduced miR-135a expression in PC-3 cells. Accordingly, we report a resistance mechanism to ADT mediated by miR-135a whose down regulation caused loss of tumor-suppressive activity that promoted PCa progression in response to ADT.

## RESULTS

### miR-135a is a direct target of AR

**Figure 1:**
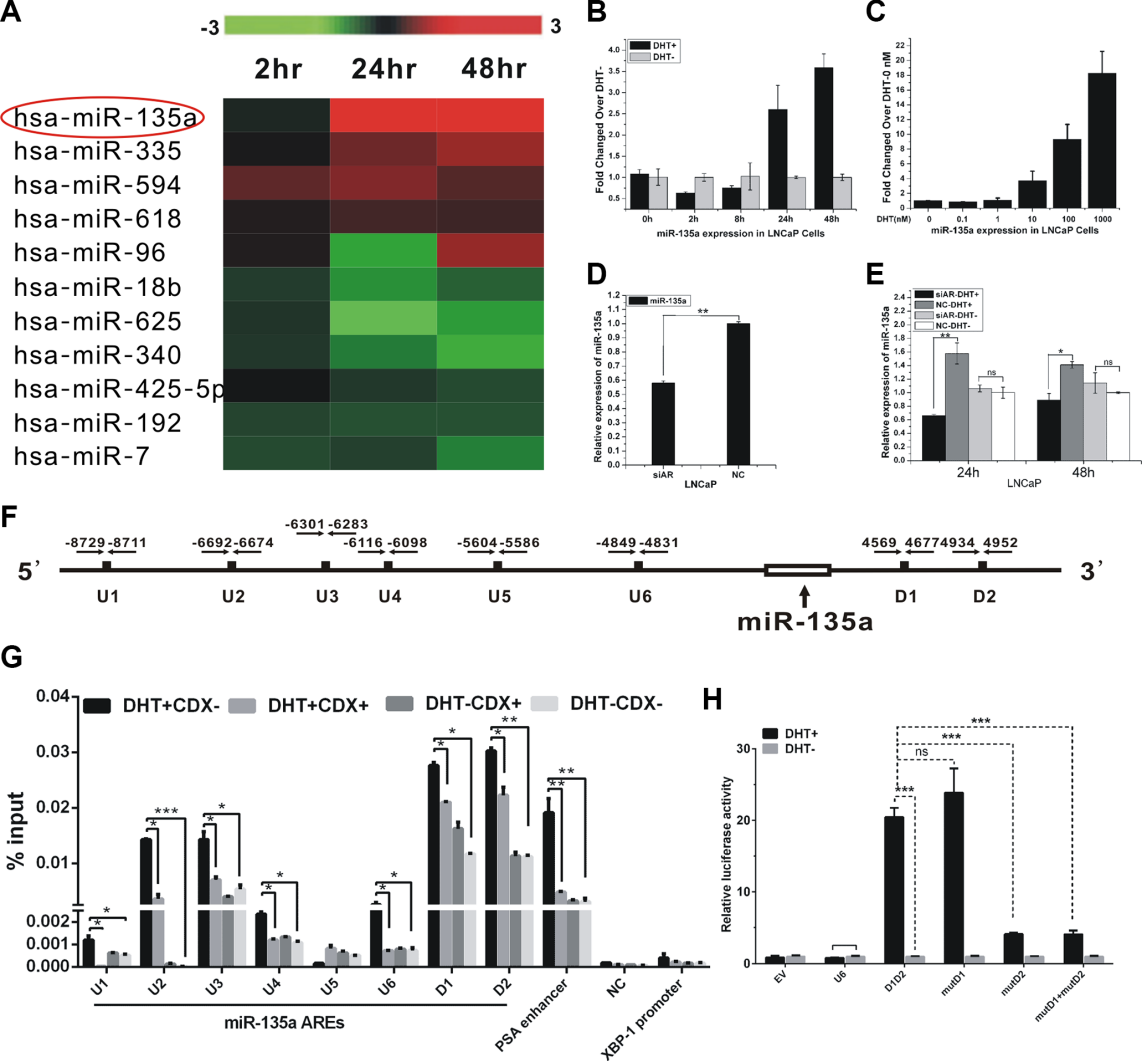
Identification of androgen-responsive miRNAs in PCa cells (**A**) Expression profile of microRNAs in LNCaP cells with androgen-starved for 72 h and treated with 10 nM DHT at different time point. Upregulated microRNAs are shown in red, while downregulated microRNAs are shown in green. (**B**–**C**) qRT-PCR analysis of miR-135a expression with androgen-starved for 72 h and treated with 10 nM DHT at different time points and doses in LNCaP. (**D**–**E**) qRT-PCR analysis of miR-135a expression in LNCaP cells with or without AR knockdown by siRNA along with or without DHT stimulation at time points of 24 h and 48 h. (**F**) Schematic locations of in silico-identified AREs in the upstream/downstream of miR-135a. (**G**) ChIP assay of AR-binding on candidate AREs of miR-135a. PSA enhancer (KLK3) serve as positive control and XBP-1 promoter and NC served as negative control. Bicalutamide (CDX) is an AR antagonist. (**H**) LNCaP cells were co-transfected with firefly luciferase reporter vector harboring indicated ARE and Renilla luciferase plasmid as an internal control. Relative luciferase activity of cells induced with DHT was compare with that treated with EtOH. EV, empty vector, was used as a negative control. Values, expressed as percentages of input DNA, are presented as the mean ± SD of at least three independent experiments. Significance was defined as *p* < 0.05 (*p < 0.05; **p < 0.01; ***p < 0.001); ns means not significant.

Dynamic expression of microRNAs in LNCaP cells treated with dihydrotestosterone (DHT) has been analysed previously [13]. From our previous data set (GSE21245), we identified additional eleven miRNAs’ expressions were significantly changed in the later period of androgen treatment (Figure 1A). Notably, miR-135a expression was greatly increased after DHT treatment. To validate whether miR-135a is androgen induced, we performed qRT-PCR experiments to monitor miRNA expression under time-course DHT treatment conditions in LNCaP cells. As shown in Figure 1B, miR-135a expression increased by 2.5 fold after 24 h and by 3.5 fold after 48 h in LNCaP cells treated with DHT, compared with the vehicle control. Furthermore, a dose-response study was also recruited to explore the expression of miR-135a under different doses of DHT (0, 0.1, 1, 10, 100 and 1000 nM). Notably, miR-135a expression levels increased more than 15 fold after high dose of DHT (1000 nM) stimulation compared with control (Figure 1C). A similar androgen-induced miR-135a expression was also observed in a dose-dependent manner and time course in 22Rv1 cells (Supplementary Figure 1A and 1B). The results showed DHT stimulation increased miR-135a expression in a dose-dependent manner.

Moreover, we observed that miR-135a expression was reduced by 40 percent over negative control (NC) following siAR transfection (Figure 1D). To further validate the regulation of miR-135a by AR, we examined miR-135a expression after siAR transfection in the presence or absence of 10 nm DHT in LNCaP cells. Our data showed that miR-135a expression was decreased by AR knockdown in the presence of DHT stimulation, but the reduction of miR-135a was not observed in the absence of DHT, suggesting that AR directly induced the androgen-mediated miR-135a expression (Figure 1E).

To prove the hypothesis that AR regulates the expression of miR-135a by directly binding to its promoter, we performed ChIP-PCR assay to verify binding of AR to putative AREs of miR-135a. Eight putative AREs (named U1–U6, D1–D2) were detected in the proximal DNA regions of miR-135a (Figure 1F) with Genomatix database [21]. By ChIP-qPCR, a significant increase of AR binding to the chromatin of putative AREs except U5 was showed in LNCaP cells treated with 100 nM DHT for 4 h only (Figure 1G). In addition, direct binding of AR to these predicted AREs was significantly reduced by CDX (bicalutamide), an AR antagonist (Figure 1G). PSA (KLK3) enhancer was served as positive control for AR-binding, and a DNA region with no putative ARE adjacent to miR-135a (named NC) and XBP-1 promoter were served as negative controls (Figure 1G). To confirm our ChIP results, we also use pGL4.23 reporter assay to identify the AR binding sites. DNA segment containing AREs (U6, D1 and D2), which are not contained in Krosis's research, are cloned in luciferase reporter system. After 24 hrs incubation, the relative luciferase expressions of D1D2 vectors were significantly up-regulated by DHT stimulation (Figure 1H). However, the relative luciferase expression of U6 vectors was not changed after DHT stimulation. Mutation of D2 significantly decreased the expression of luciferase (Figure 1H). Taken together, miR-135a was directly upregulated by AR in prostate cancer cells.

### MicroRNA-135a directly suppresses RBAK and MMP11 in prostate cancer cells

**Figure 2:**
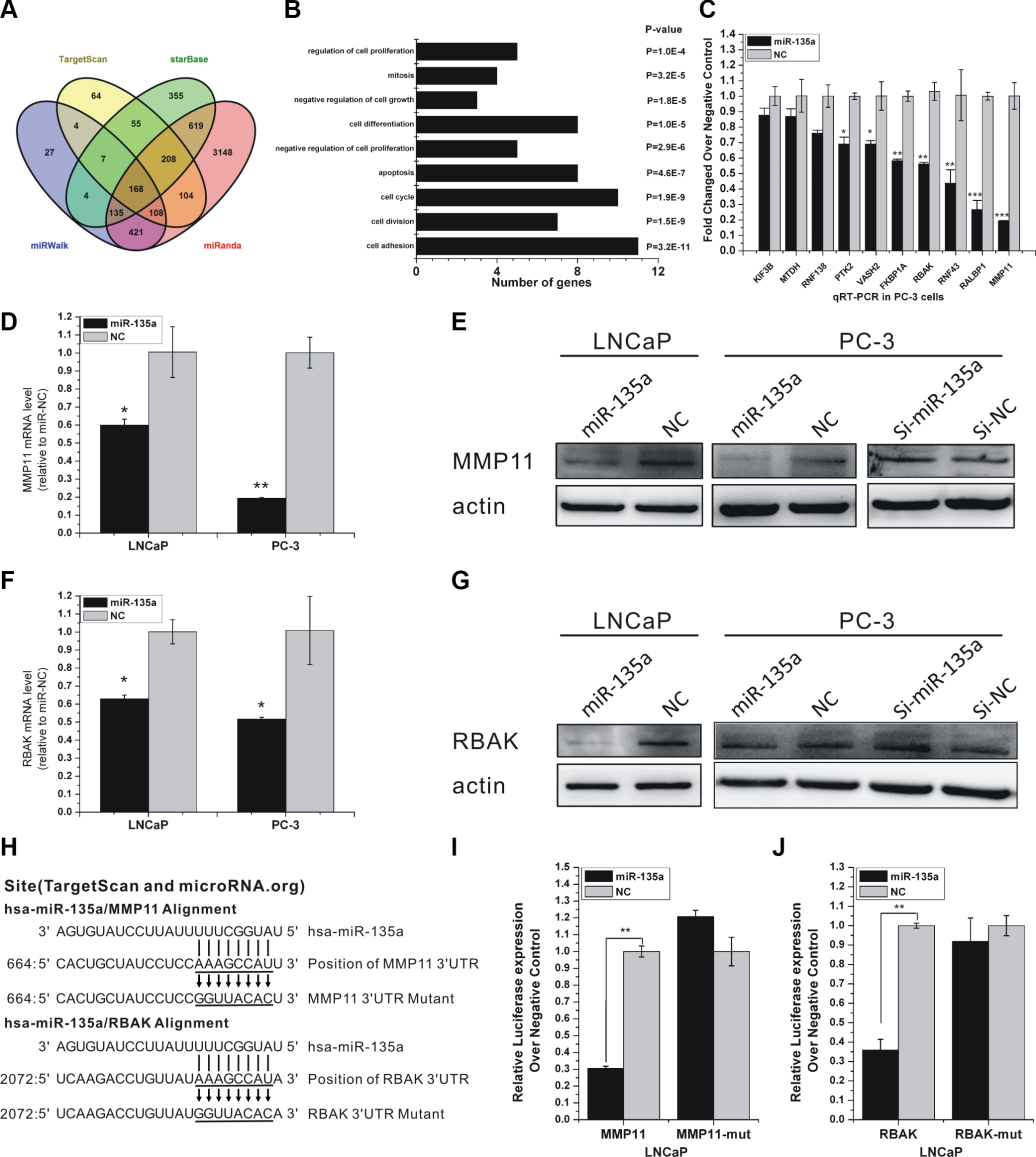
miR-135a directly targets RBAK and MMP11 in PCa cells (**A**) Venn diagrams for miR-135a targeted genes using four open-access softwares, including TargetScan, miRanda, miRwalk, and starBase. (**B**) GO analysis of predicted targets of miR-135a. Number of genes and *P* value of each GO category are indicated at x-axis and next to the bar, respectively. (**C**) RT-PCR analysis of ten miR-135a's potential target gene expressions after miR-135a mimic transfection in PC-3. (**D**–**G**) qRT-PCR and western blotting analysis of MMP11 and RBAK mRNA and protein expression levels after miR-135a mimic or miR-135a inhibitor transfection in LNCaP and PC-3 as indicated. β-actin was used as internal control. (**H**) Sequence alignment of human miR-135a and 3′-UTR of RBAK and MMP11 using TargetScan and microRNA.org. The seed sequence of miR-135a (top) matches 3′-UTR of MMP11 and RBAK (middle). Bottom is the mutations of the 3′-UTR of MMP11 and RBAK used in luciferase reporter construct. (**I**–**J**) Luciferase assay in LNCaP cells. MiR-135a over-expression decreased the luciferase activity of MMP11 and RBAK, but did not affect that of MMP11-mut and RBAK-mut. Data are presented as the mean ± SD (*n* = 3). Significance was defined as *p* < 0.05 (*p < 0.05; **p < 0.01; ***p < 0.001); ns means not significant.

To obtain insight into the potential biological process and molecular pathways affected by miR-135a, we performed bioinformatic analyses for miR-135a-targeted genes using four publicly available algorithms including TargetScan (www.targetscan.org) [22], miRWalk (http://www.umm.uni-heidelberg.de/apps/zmf/mirwalk/) [23], miRanda (http://www.microrna.org/microrna/home.do), and starBase (http://starbase.sysu.edu.cn/) [24], which resulted in 168 unique targets predicted by at least three different algorithms (Figure 2A). These genes were mapped using the Kyoto Encyclopedia of Genes and Genomes (KEGG) pathway database and BioCarta pathway database, which enabled the annotation of 168 genes. Our data revealed that miR-135a-targeted genes strongly overlapped with genes that regulated biological processes, including cell adhesion, cell division, cell cycle, and cell apoptosis (Figure 2B).

To identify putative targets of miR-135a, we performed qRT-PCR to examine the changes in expressions of ten miR-135a's targets. As shown in Figure 2C, RBAK and MMP11, which were critical attenuators of cell proliferation and cell migration, were identified as potential targets of miR-135a. Both mRNA and protein levels of RBAK and MMP11 were dramatically decreased by ectopic expression of miR-135a compared with miR-NC in LNCaP and PC-3 cells (Figure 2D–2G). Western blotting analyses also showed that inhibition of miR-135a increased the protein expression levels of RBAK and MMP11 in LNCaP and PC-3 cells (Figure 2E and 2G).

To determine whether RBAK and MMP11 are direct targets of miR-135a, we performed luciferase reporter assay with vectors containing 3′UTRs flanking the putative binding sites of miR-135a. Mutations in the putative binding sites were created as controls (Figure 2H). MiR-135a significantly decreased the luciferase activity of RBAK and MMP11 vectors compared with miR-NC (Figure 2I, 2J). However, in the mutant RBAK and MMP11 3′UTR group, no detectable change in luciferase activity was observed (Figure 2I, 2J). The result denoted that miR-135a directly downregulated the expression of these genes through their 3′UTRs.

### Overexpression of miR-135a inhibited proliferation and delayed cell cycle progression of prostate cancer cells by suppressing RBAK

**Figure 3:**
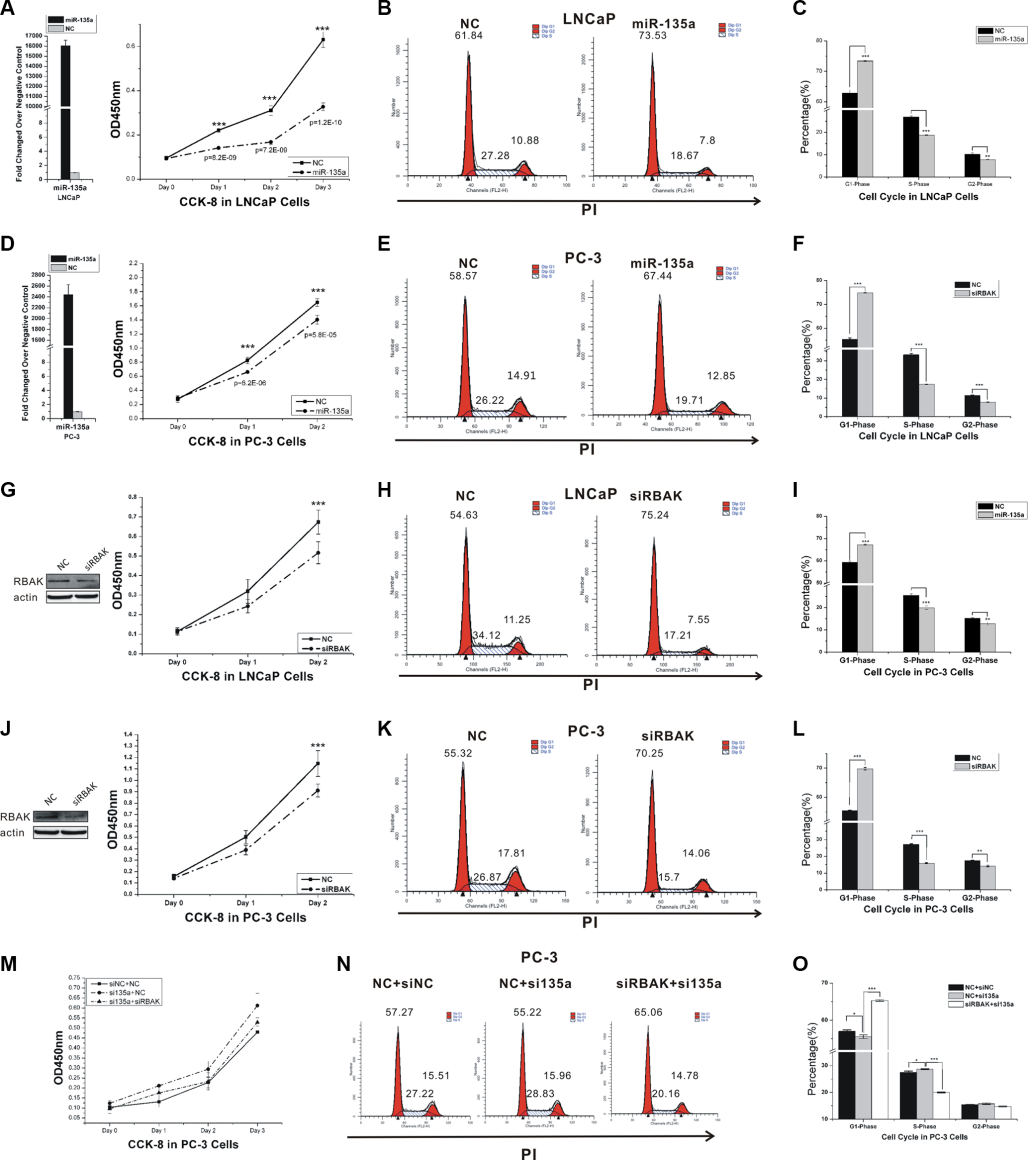
MiR-135a inhibits cell proliferation and cell cycle by targeting and suppressing RBAK (**A**, **D**, **G**, **J** and **M**) Cell proliferation analysis was performed with CCK-8 assay in LNCaP (A, G) and PC-3 cells (**B**, J and M). Cells transfected with miR-135a or siRBAK were seeded into 96-well plate at 5000 cells/well and examined at different time point. miR-135a expression was confirmed by RT-PCR (showed in the histogram) and RBAK protein knockdown was confirmed by western blot. (B, **E**, **H**, **K** and **N**) Cell cycle assay was performed in LNCaP and PC-3 cells. Cells were transfected with miR-135a or siRBAK for 48 h, stained with PI and evaluated with a FACScalibur flow cytometer. (**C**, **F**, **I**, **L** and **O**) The cell apoptosis analysis results presented as mean ± SD (*n* = 3). Significance was defined as *p* < 0.05 (*p < 0.05; **p < 0.01; ***p < 0.001); ns means not significant.

To evaluate the biological functions of miR-135a in the development of PCa, we conducted gain-of-function studies in PCa cells by transient transfection with miR-135a mimics. The CCK-8 assay indicated that overexpression of miR-135a significantly reduced the growth rate of LNCaP, PC-3 and 22Rv1 cells (*P* < 0.001, Figure 3A and 3D, Supplementary Figure 2A). As cell cycle distribution was a parameter reflecting the growth of cells, we assessed the function of miR-135a on cell cycle profile of LNCaP and PC-3 cells by flow cytometry. Overexpression of miR-135a significantly induced the increase in G1 phase and the decrease in S phase of LNCaP and PC-3 cells (Figure 3B–3C and 3E–3F).

RBAK was reported as a transcriptional repressor that interacted with RB to influence E2F mediated cell cycle regulation [25, 26]. However, little was known about RBAK in PCa until now. To evaluate the role of RBAK in regulating cell proliferation, we suppressed the expression of endogenous RBAK using specific siRNAs in PCa cell lines. Knockdown of RBAK markedly suppressed cell proliferation and induced the increase in G1 phase and the decrease in S phase of LNCaP and PC-3 cells (Figure 3G–3L).

To explore the effects of miR-135a on the growth of PCa cells by targeting and suppressing RBAK, we co-transfected RBAK siRNA together with the miR-135a inhibitor into PCa cells. Knockdown of RBAK reversed the growth promotion of PC-3 cells that was induced by the miR-135a inhibitor (Figure 3M–3O). These data suggested that miR-135a inhibited proliferation and delayed cell cycle progression of prostate cancer cells by suppressing RBAK.

### Overexpression of miR-135a induced apoptosis of PCa cells by suppressing RBAK

**Figure 4:**
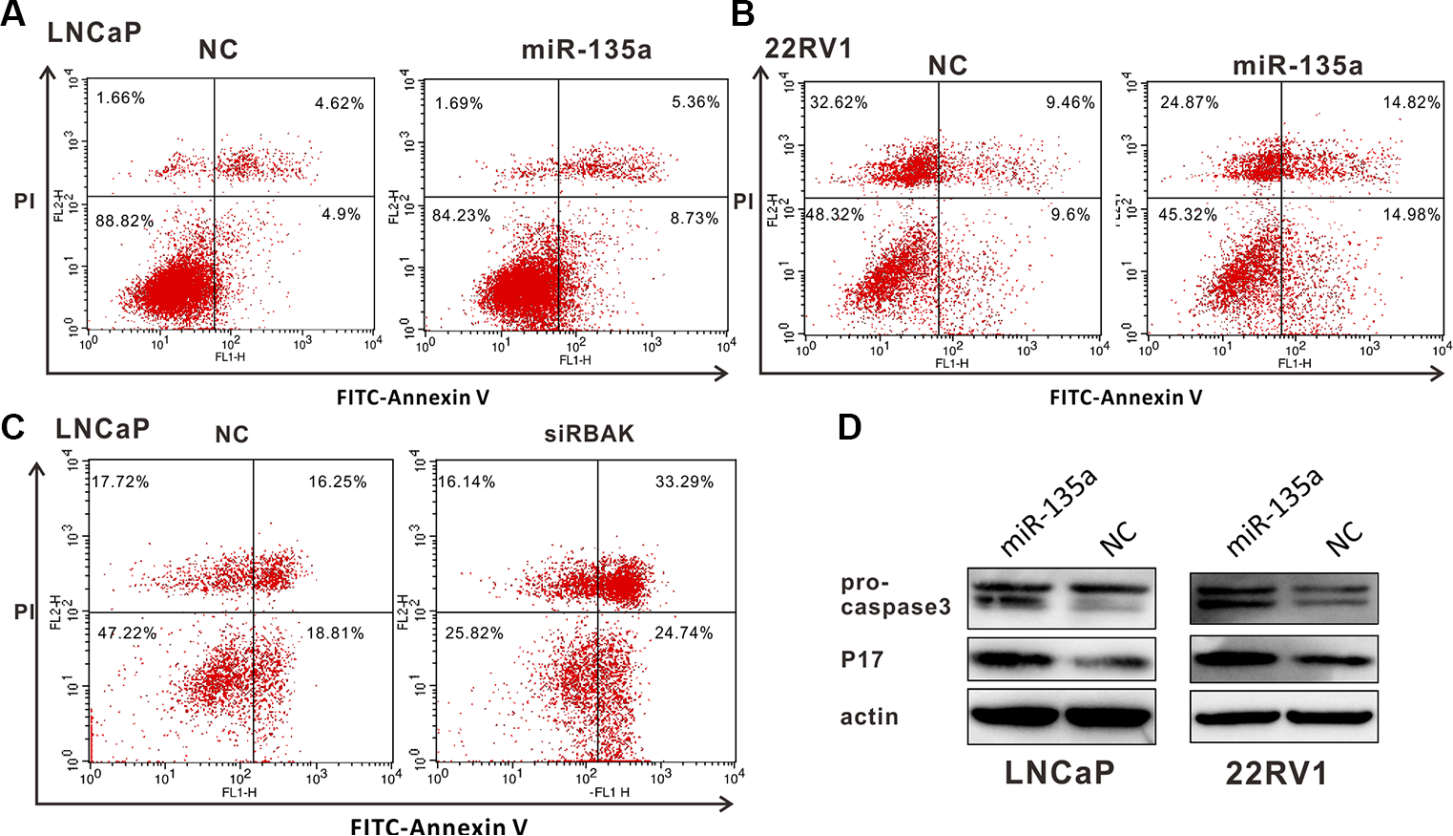
MiR-135a induced apoptosis of PCa cells by targeting and suppressing RBAK (**A**–**C**) Cell apoptosis assay was performed with flow cytometer. Cells were transfected with miR-135a or siRBAK for 48 h, and subjected to cell apoptosis assay. Both over-expression of miR-135a in LNCaP (A) /22Rv1 (B) cells and knockdown of RBAK in LNCaP cells (C) increased the fraction of both early apoptotic cells and late apoptotic cells. Data are presented as the mean ± SD (*n* = 3) in Supplementary Figure 3A–3C. (**D**) Effect of miR-135a on the protein expression levels of pro-caspase 3 and the active cleavage products of caspase-3 (P17) in LNCaP and 22Rv1 cells using western blot analysis. β-actin was used as internal control. Significance was defined as *p* < 0.05 (*p < 0.05; **p < 0.01; ***p < 0.001); ns means not significant.

We then explored the influence of miR-135a and RBAK on apoptosis of prostate cancer by flow cytometry. We found that overexpression of miR-135a in LNCaP and 22RV1 cells and silencing of RBAK in LNCaP cells increased the fraction of apoptotic cells in these PCa cell lines (*P* < 0.05, Figure 4A–4C and Supplementary Figure 3A–3C). The results revealed that RBAK functioned as an oncogene by inhibiting cell apoptosis of PCa cells, supporting the conclusion that miR-135a induced apoptosis of PCa cells, at least in part, by targeting and suppressing RBAK.

To clarify the mechanism of miR-135a affecting endothelial cell apoptosis of prostate cancer, the protein expression levels of pro-caspase3 and the active cleavage products of caspase3 (P17) were assayed by Western blot analyses. The results reflected overexpression of miR-135a promoted the cleavage of pro-caspase3 to its active 17 kDa form (Figure 4D). Besides, we used RT-PCR to detect the mRNA level of caspase3. Our results demonstrated that caspase3 were upregulated both in mRNA and in protein level (Figure 4D and Supplementary Figure 3D and 3E), and miR-135a promoted the cleavage of pro-caspase3 to its active 17 kDa form. Taken together, miR-135a induces apoptosis via activation of caspase3.

### MMP11 mediated the effect of miR-135a on PCa cells migration

**Figure 5:**
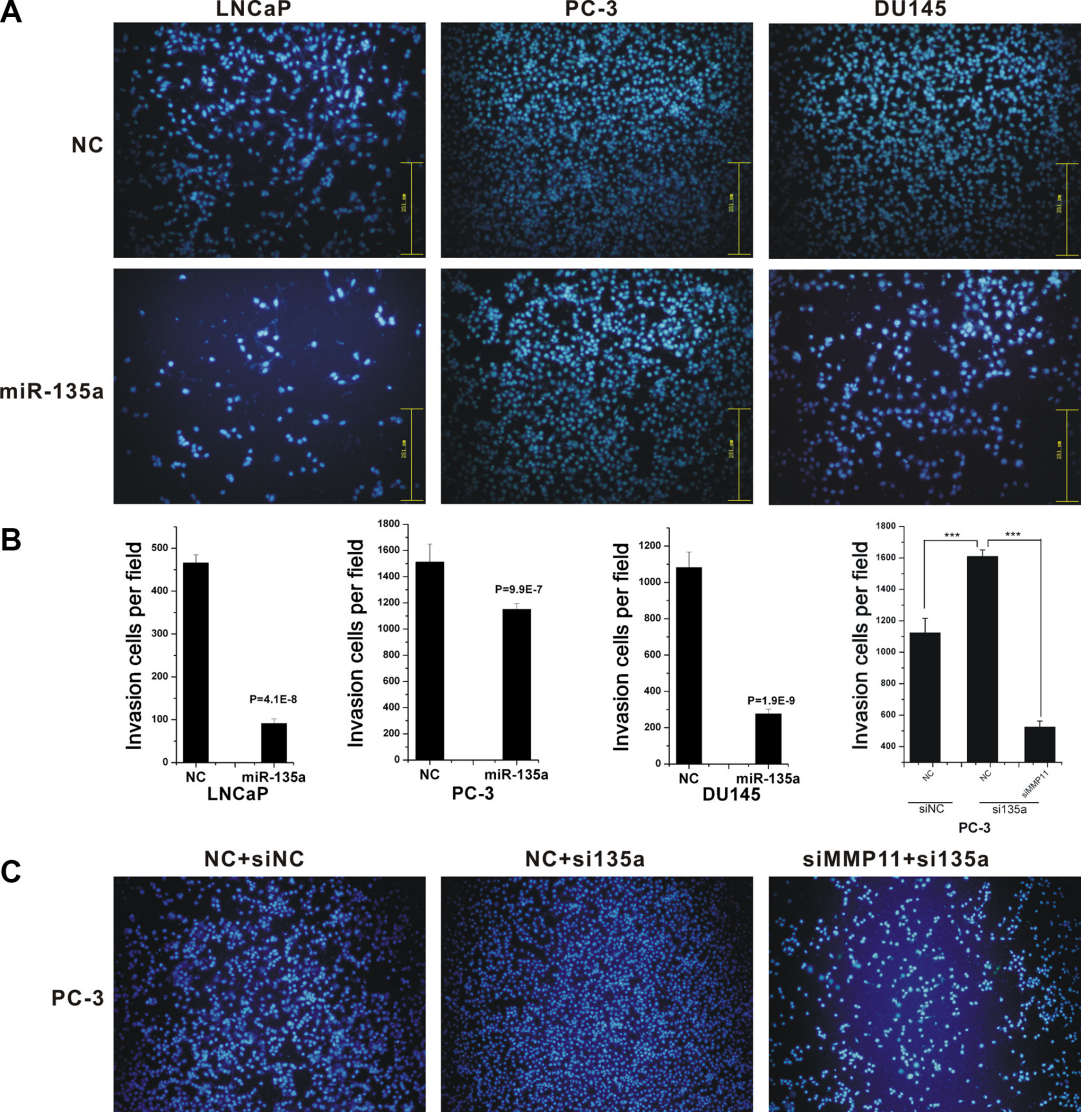
MiR-135a inhibited cell migration and invasion Migrations of LNCaP, PC-3 and DU145 cells after miR-135a transfection (**A**), and migrations of PC-3 cells after si-miR-135a transfection and si-miR-135a - siMMP11 co-transfection (**C**) were counted by transwell assay. (**B**) The cell cycle analysis results presented as mean ± SD (*n* = 3). Significance was defined as *p* < 0.05 (*p < 0.05; **p < 0.01; ***p < 0.001); ns means not significant.

To further characterize the tumor suppressive function of miR-135a, transwell migration assay was employed to detect the effect of miR-135a on PCa cells migration. As shown in Figure 5, ectopic expression of miR-135a significantly decreased the migration ability of LNCaP, DU145 and PC-3 cells by 45%, 55% and 50%, respectively, compared to negative control cells (*P* < 0.001, Figure 5A and 5B).

MMP11 was broadly reported as a key regulator of cell migration and invasion [27, 28]. As previously mentioned, we identified MMP11 as a miR-135a regulated gene, suggesting that miR-135a significantly inhibited prostate cancer cells migration via suppression of MMP11. To elucidate the suggestion, we then performed co-transfection of miR-135a inhibitor and MMP11 siRNA in PC-3 cells, and the results proved that knockdown of MMP11 significantly reversed the stimulative effect of miR-135a inhibitor on migration in PC-3 cells (Figure 5C). These data suggested that miR-135a inhibited PCa migration, at least in part, by suppressing MMP11.

### miR-135a mediated the crosstalk between AR and PI3K/AKT signaling pathways

**Figure 6:**
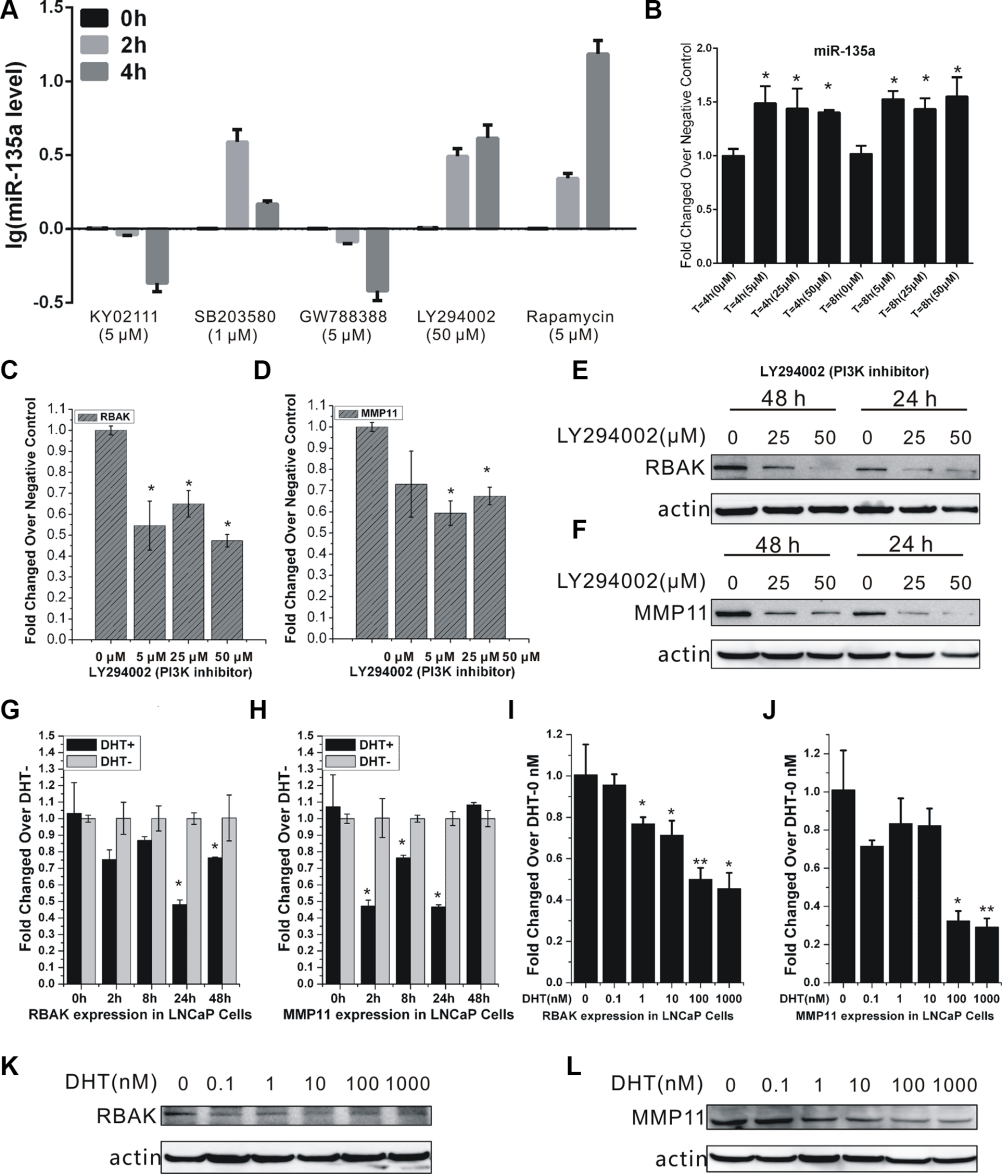
Crosstalk between AR and the PI3K/AKT pathway regulated RBAK and MMP11 expression (**A**) Detection of miR-135a expression changes in PC-3 cells after treatment with different pathway-specific inhibitors: Wnt pathway inhibitor (KY02111), MAPK pathway inhibitor (SB203580), TGF-βpathway inhibitor (GW788388), PI3K pathway inhibitor (LY294002) and mTOR pathway inhibitor (Rapamycin). (**B**) qRT-PCR analysis of miR-135a expression in PC-3 cells after treatment with the different concentration of LY294002 for 4 h and 8 h. (**C**–**D**) qRT-PCR analysis of RBAK and MMP11 expression in PC-3 cells after treatment with LY294002 in different concentration for 4 h. (**E**–**F**) Western blot analysis of RBAK and MMP11 expression in PC-3 cells after treatment with LY294002 in different concentration for 24 h and 48 h. (**G**–**J**) qRT-PCR analysis of RBAK and MMP11 expression in LNCaP cells after treatment with or without DHT in different dose and time points for 4 h. (**K**–**L**) Western blot analysis of RBAK and MMP11 after treatment with DHT in different concentration for 48 h. Values of dose 0 nM of each time point were used as control for that time point dose-dependent treatment. Data are presented as the mean ± SD (*n* = 3). Significance was defined as *p* < 0.05 (*p < 0.05; **p < 0.01; ***p < 0.001); ns means not significant.

Besides AR signaling, recent evidences suggest other signaling pathways involved in prostate cancer development and progression [29]. Several findings have illustrated that PI3K/AKT/mTOR pathway upregulation resulting from PTEN loss is associated with the development of CRPC [30–32]. Emerging evidence demonstrates a key role for the PI3K/AKT/mTOR signaling axis in the development and maintenance of CRPC [33]. In this study, to further explore the potential pathways regulating miR-135a expression in androgen-independent PCa cells, we screened a series of pathway-specific inhibitors in PC-3 cells and found that PI3K/AKT pathway inhibitors LY294002 and mTOR pathway inhibitor Rapamycin could induce miR-135a expression in a time-dependent manner (Figure 6A). As mTOR pathway is a downstream of PI3K/AKT pathway, these results suggested that miR-135a might be regulated by PI3K/AKT signaling. The following study also showed that LY294002 could induce miR-135a expression and down regulate RBAK and MMP11 mRNA and protein level simultaneously in a dose-dependent manner (Figure 6B–6F), indicating that PI3K/AKT signaling was involved in regulation of miR-135a expression in androgen-independent PCa cells.

As previously mentioned, we identified miR-135a as a direct target of AR. Thus, we reasoned that AR signaling may inhibit RBAK and MMP11 expression via inducing miR-135a expression in LNCaP. To test this hypothesis, we detected RBAK and MMP11 expression after DHT stimulation and found that their mRNA transcripts were markedly suppressed by DHT in a time-and dose-dependent manner in LNCaP cells (Figure 6G–6J). Moreover, we observed DHT significantly reduced the expression of RBAK and MMP11 protein levels in a dose-responsive manner (Figure 6K and 6L).

### MiR-135a is inversely correlated with RBAK and MMP11 in PCa tissues

**Figure 7:**
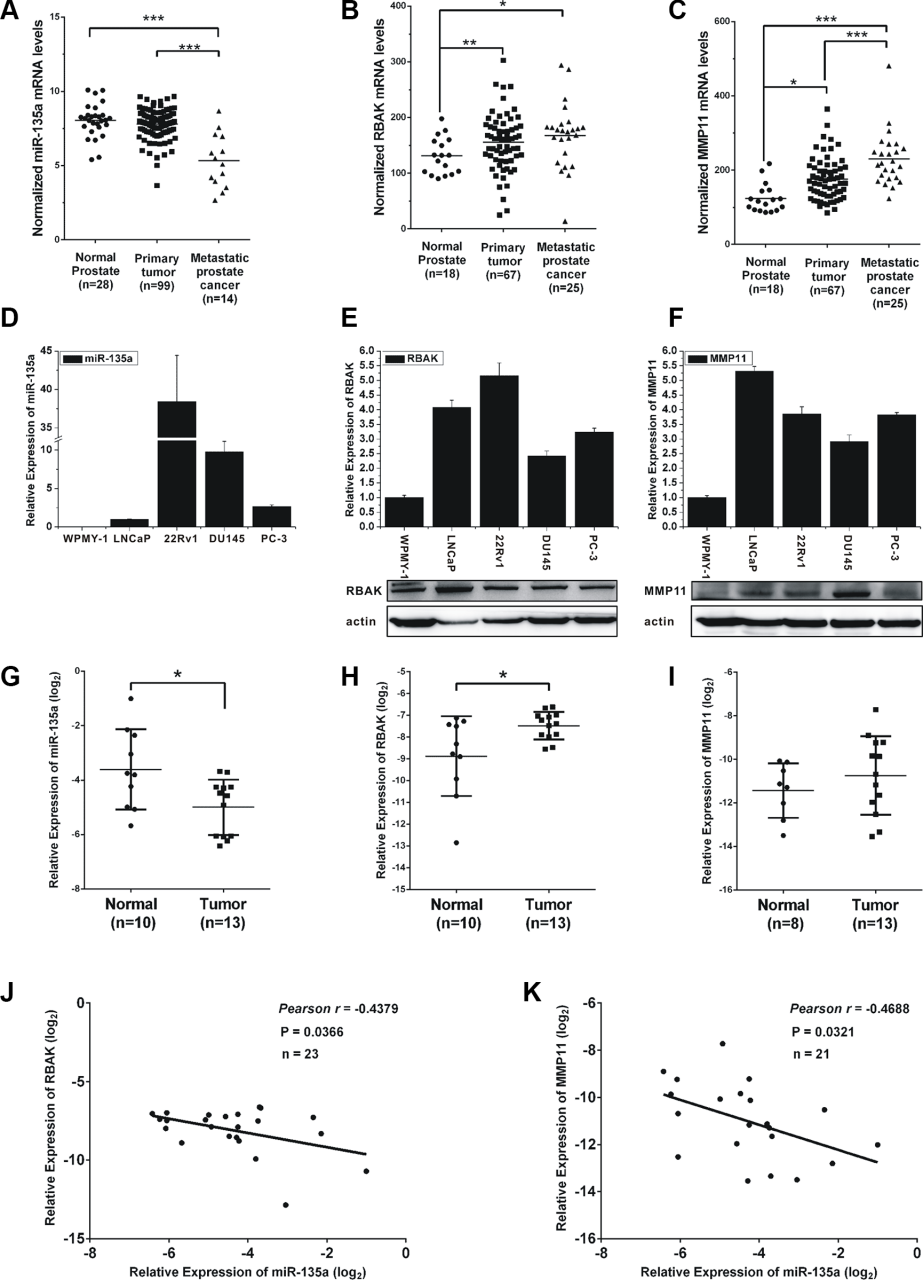
Expression of miR-135a, RBAK and MMP11 in prostate cancer cell lines and tissue samples (**A**–**C**) mRNA levels of miR-135a, RBAK and MMP11 in metastatic prostate cancers and local prostate cancers compared to normal prostate tissues by analyzing GSE21036 and GSE6919 database. (**D**) qRT-PCR analysis of miR-135a expression level in normal prostate epithelial cells line WPMY-1 and prostate cancer cell lines LNCaP, 22Rv1, DU145 and PC-3. (**E**–**F**) qRT-PCR and western blotting analysis of RBAK and MMP11 mRNA and protein expression levels in different prostate cell lines as indicated. β-actin was used as internal control. (**G**–**I**) qRT-PCR analysis of miR-135a, MMP11 and RBAK mRNA levels in normal tissues and tumor samples. (**J**–**K**) Correlation plots of miR-135a and RBAK or MMP11 by Pearson's product-moment correlation coefficient. Data are presented as the mean ± SD (*n* = 3). Significance was defined as *p* < 0.05 (*p < 0.05; **p < 0.01; ***p < 0.001); ns means not significant.

To determine the role of miR-135a and its targets in the pathogenesis of PCa, we investigated miR-135a, RBAK and MMP11 expression levels in prostate tumors compared to normal prostate tissues using two publicly available gene expression database: GSE21036 [34] and GSE6919 [35]. Analysis of GSE21036 database showed that miR-135a was significantly decreased in metastatic prostate cancers compared with local prostate cancers and normal prostate tissues (*P* < 0.001, Figure 7A). However, our results showed no significant difference of miR-135a expression between normal and primary tumor, although the mean levels of normal samples (8.045) were higher than tumors (7.800). Meanwhile, we found the mRNA levels of RBAK and MMP11 significantly increased (*P* < 0.001, Figure 7B and 7C) in metastatic prostate cancers and local prostate cancers compared to normal prostate tissues by analyzing GSE6919 database.

We then evaluated miR-135a, RBAK and MMP11 expression level in the noncancerous prostatic cells WPMY-1 and four prostate cancer cell lines LNCaP, 22Rv1, DU145 and PC-3. As shown in Figure 7, both the mRNA and protein levels of RBAK and MMP11 were significantly increased in human PCa cell lines compared with noncancerous prostatic cells WPMY-1 (Figure 7E and 7F). However, no expression of miR-135a was observed in WPMY-1 cells, and 22Rv1 cells had the highest expression of miR-135a (Figure 7D). This result was not consistent with that in PCa samples. The reason for this may be that long term culture of these cell lines could not totally reflect gene expression *in vivo* situations [36].

Next, the expression levels of miR-135a, RBAK and MMP11 were further examined in 10 normal prostate tissues and 13 prostate cancers with real-time PCR. We found that miR-135a was downregulated and RBAK was upregulated in prostate cancer samples compared with normal prostate tissues (Figure 7G–7I). The expression of MMP11 was just slightly over-expressed in tumors, possibly owing to the small sample size. Then, we correlated miR-135a with RBAK and MMP11 expression in the same 23 samples. The results showed that the RBAK and MMP11 mRNA levels were inversely correlated with miR-135a expression levels in PCa patients (*P* < 0.05, Figure 7J and 7K). These results demonstrated the miR-135a was downregulated and its target genes were upregulated in PCa patients.

### Low miR-135a and high RBAK and MMP11 levels were positively correlated with PCa progression

**Figure 8:**
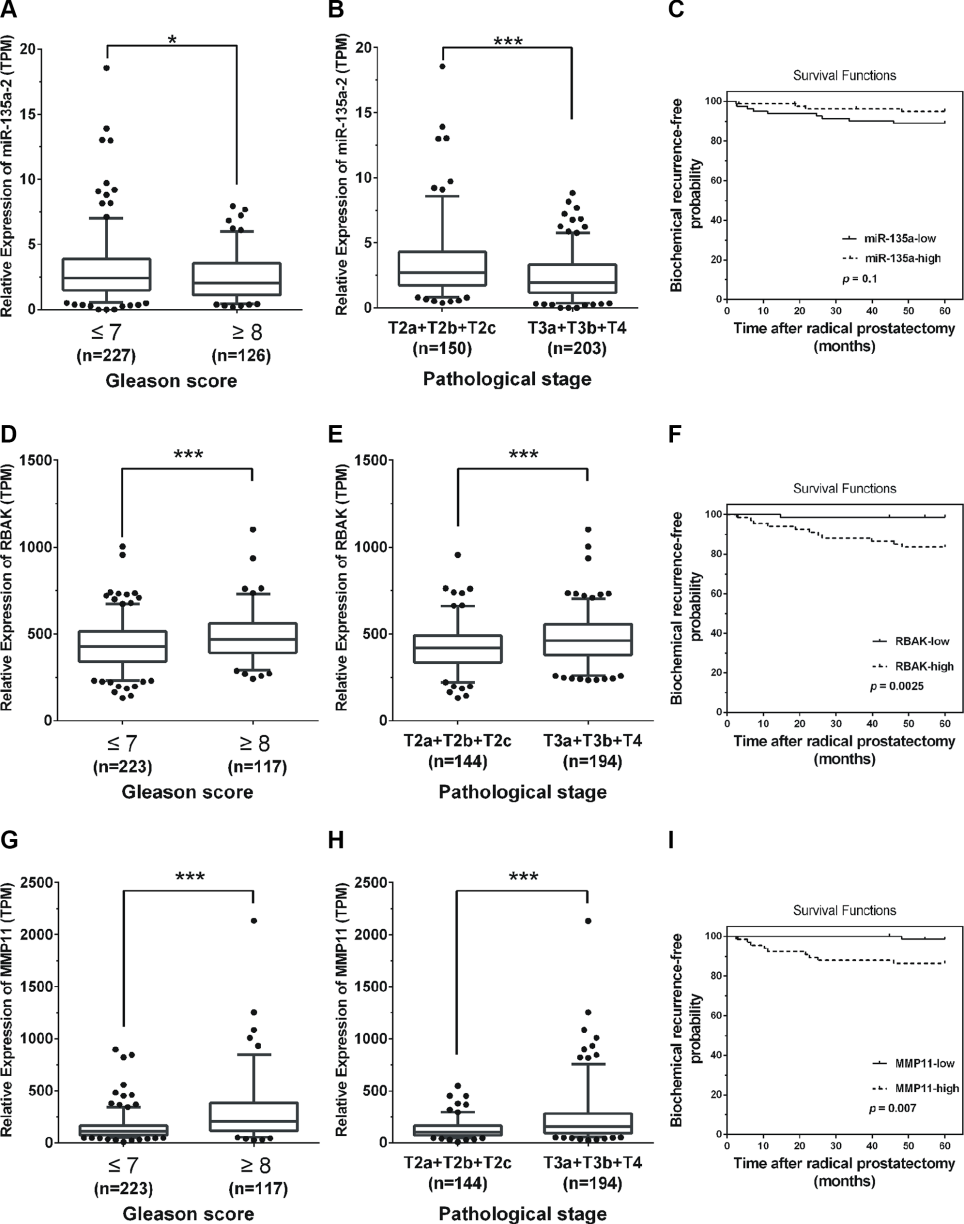
Low miR-135a and high RBAK and MMP11 levels were positively correlated with PCa progression (**A**, **B**, **D**, **E**, **G**, **H**) MiR-135a, RBAK and MMP11 expression in PCa samples in correlation with Gleason scores and pathological stages from TCGA database, respectively. (**C**, **F** and **I**) Kaplan-Meier curves for survival time after radical prostatectomy in patients with prostate cancer according to expression of miR-135a (C), RBAK (F), MMP11 (I). Significance was defined as *p* < 0.05 (*p < 0.05; **p < 0.01; ***p < 0.001); ns means not significant.

To evaluate possible prognostic value of miR-135a, RBAK and MMP11, we analyzed RNAseq data from TCGA, a cohort of 52 normal prostate tissues and 419 prostate cancer samples. As shown in Figure 8A, miR-135a was expressed at lower levels with a high Gleason score (≥ 8), compared with tumors with Gleason score ≤ 7. Furthermore, the expression level of miR-135a was statistically decreased in invasive extraprostatic tumors (pT3a, pT3b and T4 stages) as compared with intraprostatic localized tumors (pT2a, pT2b and pT2c stages) (Figure 8B). Kaplan-Meier analysis and the log-rank test indicated that a high level of miR-135a expression was slightly associated with lower biochemical recurrence (*P* = 0.1; Figure 8C). Our data are consistent with previous studies.

We then analyzed the relation between RBAK and MMP11 expression and pathological grading. As shown in Figure 8D and 8G, RBAK and MMP11 were expressed at higher levels with a low Gleason score (≤ 7), compared with tumors with Gleason score ≥ 8. Furthermore, the expression level of RBAK and MMP11 were statistically increased in invasive extraprostatic tumors (pT3a, pT3b and T4 stages) as compared with intraprostatic localized tumors (pT2a, pT2b and pT2c stages) (Figure 8E and 8H). Next, we also used a Kaplan-Meier analysis to evaluate whether RBAK and MMP11 expression were associated with patient outcome. As expected, we found that a high level of RBAK (*P* = 0.0025) and MMP11 (*P* = 0.007) were associated with significantly lower biochemical recurrence (Figure 8F and 8I). Taken together, these results indicated that the low level of miR-135a and the high levels of RBAK and MMP11 were correlated with a short biochemical recurrence-free survival times.

## DISCUSSION

The main challenge in the management of prostate cancer was the development of resistance to androgen deprivation therapy (ADT). Therefore, identifying new molecules and signals linked to hormone independent behavior of PCa was of great importance. In the present study, we demonstrated that as an androgen-induced tumor suppressor, miR-135a decreased PCa cells proliferation and induced PCa cell cycle arrest and apoptosis through its target RBAK, and repressed cell migration by down-regulating the expression of MMP11. Furthermore, PI3K/AKT pathway was confirmed to be an upstream regulation signaling of miR-135a in androgen-independent cell lines. Moreover, our results showed low level of miR-135a and high level of RBAK and MMP11 were positively correlated with PCa progression.

The molecular mechanisms involved in the androgen-resistance transformation of PCa remained unclear. Emerging evidence had indicated that AR signaling plays a critical role in it [5]. Deregulation of miRNAs was involved in tumor formation and progression of different cancers. Herein, we identified miR-135a as a candidate microRNA by our previous microarray analysis. Coincidently, Kroiss and his colleagues also identified miR-135a as an androgen upregulated miRNA [15]. Besides, we showed that miR-135a expression was greatly upregulated after DHT stimulation and was downregulated after AR knockdown. Direct regulation of miR-135a expression by AR was supported by ChIP assay. A significant increase of AR binding to several putative AREs of pre-miR-135a was detected in LNCaP cells treated with 100 nM DHT. More importantly, direct binding of AR to these predicted AREs were identified by luciferase reporter assay. Our data are consistent with previous studies that the expression of miR-135a was regulated by AR.

Previous researches had illustrated that miR-135a was abnormally expressed in various tumors and involved in various biological processes, such as cell proliferation, migration and metastasis [17, 18, 37, 38]. However, it was not well described that the complex biologic processes mediated by miR-135a which inhibited tumor proliferation. To explore the function of miR-135a on prostate cancer, we overexpressed miR-135a in PCa cells and found that enhanced miR-135a expression could significantly decrease PCa cells proliferation and migration, and induce PCa cells cycle arrest and apoptosis. Furthermore, we observed lower expression of miR-135a in prostate cancer samples compared with normal prostate tissues by analyzing publicly available gene expression data and examining tissues expression levels using RT-PCR. Taken together, these results suggested that miR-135a acted as a tumor suppressor whose down-regulation contributed to the progression and metastasis of PCa.

In the present study, we identified RBAK and MMP11 as direct targets of miR-135a in PCa cells by integrating bioinformatics analysis and experimental assays. Overexpression of miR-135a reduced RBAK and MMP11 both mRNA and protein levels in PCa cells. Moreover, miR-135a is inversely correlated with RBAK and MMP11 in PCa tissues. In this study, we confirmed that RBAK was an oncogene and mediated the effect of miR-135a on PCa cells proliferation, which was consistent with previous report. Intriguingly, RBAK was reported as a co-factor that interacted with the androgen receptor and enhanced its transcriptional activity, suggesting the existence of a feedback loop. MMP11 was overexpressed in numerous types of human carcinoma [39, 40], including breast [41], non-small cell lung, gastric cancer [42], colorectal carcinomas [43, 44] and prostate cancer [45]. Overexpression of MMP11 could promote invasion of gastric cancer cells [42]. In this study, we found knockdown of MMP11 significantly inhibited PCa cell migration. Our research confirmed that miR-135a inhibited migration of PCa cells, at least in part, by suppressing MMP11. In prostate cancer, Kroiss and his colleagues also demonstrated that miR-135a decreased prostate cancer cell migration and invasion through downregulating ROCK1 and ROCK2 [15]. ROCK is an effector of the small GTPase Rho and belongs to the AGC family of kinase. ROCK had emerged as a family of protein kinases that regulate the process of plasticity during cancer cell migration [46–48]. Moreover, previous reports showed that RhoA/ROCK pathway was upstream of MMPs. Taken together, our results together with Kroiss’ report highlighted the important role of miR-135a in regulating cell migration via ROCK1, ROCK2 and MMP11.

The main aim of this research was to explore the mechanism of CRPC. Besides AR signaling, recent studies suggest that other signaling pathways are involved in the evolution of CRPC, such as PI3K/AKT/mTOR pathway, RAS/MAPK pathway and Wnt/β-catenin pathway [3, 5]. In this study, we screened a series of pathway-specific inhibitors to explore the potential pathways regulating miR-135a and found that PI3K signaling down-regulated miR-135a expression in PC-3 cells. In prostate cancer, several findings have illustrated that PI3K/AKT/mTOR pathway upregulation resulting from PTEN loss is associated with the development of CRPC [30–32]. Although detailed mechanisms of miR-135a regulation by PI3K/AKT signaling needs further study, our results support a model whereby PI3K/AKT hyperactivation could down-regulate miR-135a expression, therefore up-regulating RBAK and MMP11, to accelerate the progression of CRPC.

Collectively, our data suggested that downregulation of miR-135a caused loss of tumor-suppressive activities that promoted the progression of CRPC in response to ADT. Our study highlights miR-135a was an androgen-induced tumor suppressor that might be downregulated by androgen depletion, thus up-regulating RBAK and MMP11 promote PCa progression. Taken together, ADT down-regulated androgen-induced miR-135a, which was also inhibited by PI3K/AKT hyperactivation. Downregulation of miR-135a caused loss of tumor-suppressive activities that promoted the progression of CRPC in response to ADT. These findings may help us understand the molecular function of miR-135a in prostate tumorigenesis and provide a new diagnostic and therapeutic biomarker for castration-resistant PCa.

## MATERIALS AND METHODS

### Cell culture

LNCaP cells were purchased from the American Type Cult. Collection (Manassas, USA) which were confirmed by short tandem repeat (STR) analysis. 22RV1, DU145, PC-3 and WPMY-1 were obtained from Cell Bank of Chinese Academy of Sciences (Shanghai, China) where they were authenticated by mycoplasma detection, DNA-Fingerprinting, isozyme detection and cell vitality detection. All experiments were carried out with cell lines at passages below 30. The four prostate cancer cell lines were maintained in RPMI 1640 medium (Corning, USA) supplemented with 10% FBS (Hyclone, USA) and WPMY-1 in DMEM medium (Corning, USA) with 10% FBS, and they all cultured at 37°C in 5% CO_2_.

Androgen treatment assay was performed as described previously [49]. Briefly, LNCaP cells were cultivated in Phenol Red-free RPMI 1640 (GIBCO/BRL) supplemented with 10% charcoal-dextran-stripped FBS for 3 days before androgen treatment, then were induced with DHT at concentration of 10 nM. The genome-wide dynamic response to DHT was analyzed at five time points − 0 h, 2 h, 8 h, 24 h and 48 h, where ‘0 h’ represents the state before androgen action.

### RNA interference and transient transfection

Synthetic miR-135a mimic (miR-135a) and its scrambled control miRNA (miR-NC) were purchased from GenePharma (Shanghai, China), and used at the concentration of 50 nM. MMP11 siRNA (siMMP11), RBAK siRNA (siRBAK) and its negative control (siNC) were from Biotend Biological technology (Shanghai, China), and used at the concentration of 100 nM. All sequences of synthetic oligonucleotides are listed in Supplementary Table 1. Transfection was carried out with Lipofectamine 2000 Transfection Reagent (Life, USA) according to the manufacturer's procedure.

### RNA isolation and real-time qPCR

qRT-PCR for mRNAs was performed as described previously. Specific primers for mature miR-135a were from GenePharma (Shanghai, China). Primers used for qRT-PCR were listed in Supplementary Table 1. The Ct values were normalized using β-actin or RNU6 as internal control to estimate the different expression of genes. Relative mRNA expression was calculated using the 2^−ΔΔCt^ method. Each sample was run in triplicate to ensure quantitative accuracy.

### Chromatin immunoprecipitation (ChIP) assay

ChIP was performed as described previously. Chromatin immunoprecipitates for proteins were amplified by quantitative PCR, normalized to input, and calculated as percentages of inputs. Ethanol and dimethyl sulphoxide (DMSO) were used as vehicles of dihydrotestosterone (DHT) and casodex (CDX), respectively. LNCaP cells were treated with DHT (100 nM), CDX (10 μM), DHT-CDX combination, or vehicles for 4 h before harvesting. DNA fragments were purified and analyzed by real-time PCR. The analyses of PSA enhancer (KLK3) was served as positive controls, whereas a DNA region with no putative ARE adjacent to miR-135a and XBP-1 promoter were served as a negative control. ARE positions relating to transcription start sites, primers validating AREs and primers amplifying control genes are provided in Supplementary Table 1.

### Western blotting analysis

Cells were lysed in RIPA buffer (Boston Bioproducts) supplemented with protease inhibitors (Complete, EDTA-free; Roche Diagnostics) and PMSF (Calbiochem). Lysates were separated on a 12% acrylamide gel and subjected to Western blot analysis. Immunoblots were incubated over night at 4°C with the following primary antibodies: anti-RBAK (mouse monoclonal; MBL; 1:2, 000), anti-MMP11 (Rabbit polyclonal; Cell Signaling Technologies; 1:1, 000), anti-pro-caspase 3, P17 (Proteintech, UK), and anti-Actin antibodies (goat polyclonal ; Santa Cruz Biotechnology; 1:2, 000). Goat anti-mouse IgG-HRP and goat anti-rabbit IgG-HRP (Sigma–Aldrich, USA) secondary antibodies were used to visualize bands using Amersham ECL Prime (GE Healthcare, UK). Signal intensity of Western blots was quantified by Quantity One Software (Bio-Rad, USA).

### Reporter constructs and luciferase assay

517-bp and 671-bp nucleotide sequences corresponding to portion of the 3′-UTR of RBAK and MMP11, respectively, including the conserved predicted binding site (seed sequence) for miR-135a, were inserted into psi-CHECK2 Dual-Luciferase miRNA Target Expression Vector (Promega, USA) within the XhoI/ NotI sites. Mutagenesis was performed using Mut Express^®^ II Fast Mutagenesis Kit V2 (Vazyme, USA). All insertions were verified by sequencing. The relative luciferase activity was measured by Dual-Luciferase Reporter Assay System (Promega, USA) 48 h after transfection. (Primer sequences show in Supplementary Table 1).

### Cell proliferation assay

Cell proliferation was assessed by Cell Counting Kit-8 (CCK-8, Dojindo Laboratories, Japan) in octuplicate according to the manufacturer's instructions. Absorbance was measured at 450 nm with Microplate Reader ELx808 (Bio-Tek, USA). The absorbance at 630 nm was used as a reference.

### Cell cycle and apoptosis assay

Cells were harvested 48 h after transfection. For cycle assay, cells were incubated with 0.03% triton X-100 and propidium iodide (PI) (50 ng/mL) for 15 min; the percentages of cells in different phases of cell cycle were measured with a FACScalibur flow cytometer (BD, USA) and analyzed with ModFit software (Verity Software House, USA). For apoptosis assay, cells were assayed with FITC Annexin V Apoptosis Detection Kit (BD, USA) and analyzed by flow cytometry.

### Cell migration assay

Cells (2 × 10^4^ / well) after transfection were seeded with media containing 1% FBS into the upper chamber of transwell filter (8-μm pore size, 6.5-mm diameter; Corning, USA) on a 24-well plate. Medium containing 10% FBS was used as attractant and added to the lower well of the plate. After 120 hours of incubation, cells on the upper side of the filters were removed and cells migrated to the lower side were fixed with methanol, stained with Giemsa stain, and counted under a microscope. Cells in five randomly chosen fields per transwell filter were counted to quantify the average number of migration. Each experiment was repeated three times.

### Tissue collection

The trial was approved by the Research Ethics Committee of Tongji Hospital and verbal consent was obtained from all patients. Thirteen tumor tissues and ten adjacent normal tissues were used for an extra evaluation by qRT-PCR. All samples were collected from Tongji Hospital, a subsidiary of Shanghai Tongji University, between January 2001 and December 2013. The prostate cancer patients whom the tissues were obtained from underwent radical prostatectomy and did not receive any pre-operation treatment. The histopathological features of tumor specimens were classified according to the Gleason score system and 2002 TNM classification system.

### Statistical analysis

The numerical data were presented as mean ± standard deviation (SD) of at least three determinations. Statistical comparisons between groups of normalized data were performed using *T*-test or Mann–Whitney *U*-test according to the test condition. A *p* < 0.05 was considered statistical significance with a 95% confidence level.

## SUPPLEMENTARY FIGURES AND TABLES


