# Supplementary material for: Androgen-induced miR-135a acts as a tumor suppressor through downregulating RBAK and MMP11, and mediates resistance to androgen deprivation therapy
Source: Oncotarget. 2016 Jun 14;7(32):51284–300. doi: 10.18632/oncotarget.9992 (PMC5239475; doi:10.18632/oncotarget.9992)
Supplement: Supplementary file 1 [file oncotarget-07-51284-s001.pdf]

Androgen-induced miR-135a acts as a tumor suppressor through downregulating RBAK and MMP11, and mediates resistance to androgen deprivation therapy

Supplementary Materials

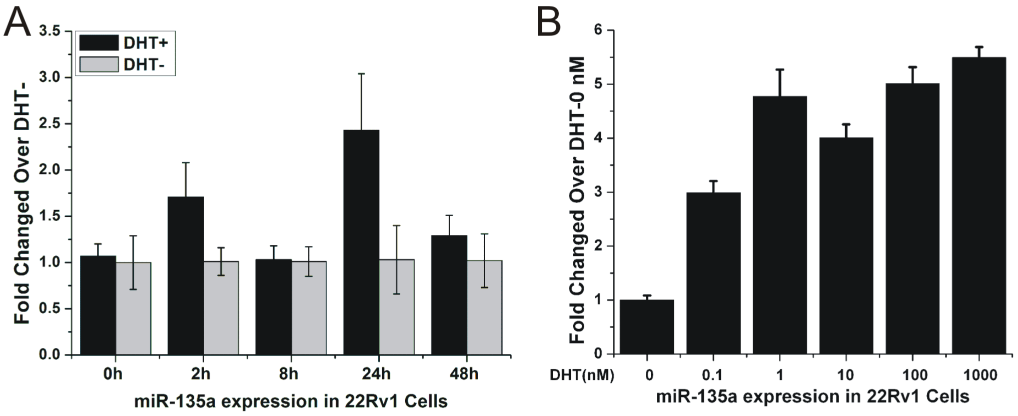

Supplementary Figure S1: qRT-PCR analysis of miR-135a expression with androgen-starved for 72 h and treated with 10 nM DHT at different time points (A) and doses (B) in 22Rv1. Significance was defined as $p < 0.05$ (*$p < 0.05$; **$p < 0.01$; ***$p < 0.001$).

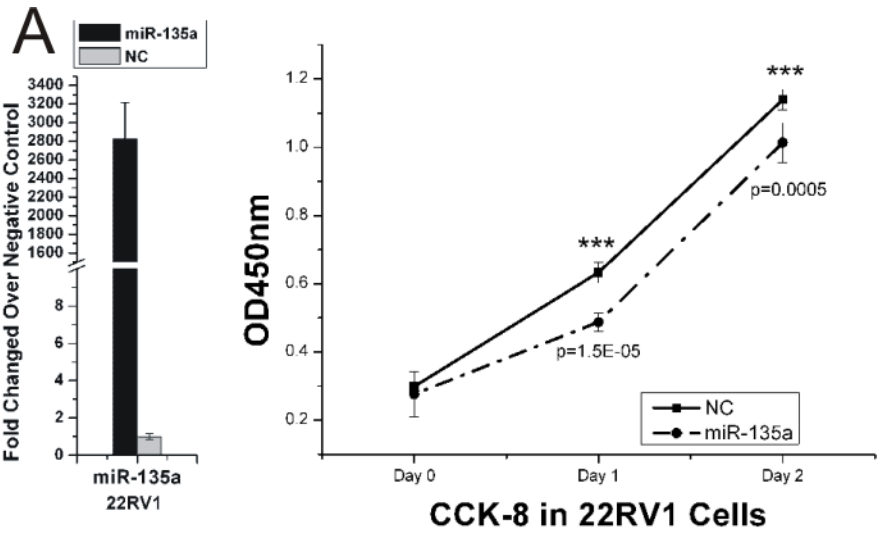

Supplementary Figure S2: Cell proliferation analysis was performed with CCK-8 assay in 22Rv1. (*$p < 0.05$; **$p < 0.01$; ***$p < 0.001$).

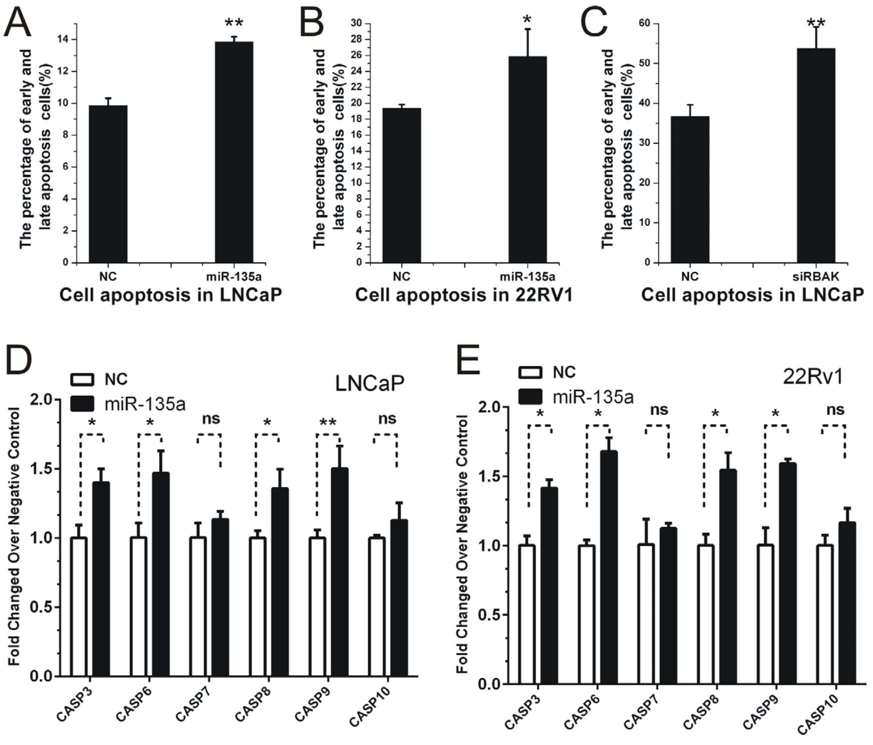

Supplementary Figure S3: RT-PCR was used to detect CASP3, CASP6, CASP7, CASP8, CASP9 and CASP10 in LNCaP and 22Rv1 cells. The cell apoptosis analysis results presented as mean ± SD ($n = 3$). Significance was defined as $p < 0.05$ (*$p < 0.05$; **$p < 0.01$; ***$p < 0.001$).

Supplementary Table S1: Primers used for ChIP-PCR, RT-PCR, clone, mutation and transfection

	Forward	Reverse
KIF3B	AGGCCCAGCTGGAAAAAC	ACCTTCCCTCCGCTTCTCT
MTDH	AGTTGCTTGGTCCCACTAGC	GCCCATGACAACCCAGATAG
RNF138	CATTTTCTTTCAGTTACATGGGTGT	AACTGGAAAACACTATGGTCAAAA
PTK2	TGAGGGAGAAGTATGAGCTTGC	TTGGCAAATAACGAATTCTCAA
VASH2	AACAGATGTTATTTCAGTCTCAGTGC	TTCCTTCCTATAAACACCTTCAGTC
FKBP1A	TCCCTCTGCTGATCTCAGTTT	AGGACAGCAAAGGGGTGAG
RBAK	CCACCAGTAAGAAAATGAACAGG	TTGTCACATACATGAACTGCAAA
RNF43	TGATTTTGGATTTTCTGTAAGTGTTT	CAGAAATTTACTCCGGGATGA
RALBP1	CTGGGTCTGCTGTCTGAGC	AGGCCAGTGGACTTGTGTG
MMP11	TCCTGAGGTCAGGTCTTGGT	CAGATTTCCAGGATTGTCAGC
siNC	UUCUCCGAACGUGUCACGUdTdT	ACGUGACACGUUCGGAGAAdTdT
siMMP11	CCUUUACUGAGGUGCACGAdTdT	UCGUGCACCUCAGUAAAGGdTdT
siRBAK	AUGUGUAAUCAGAGUAGACdTdT	CUGCUUGUAUCAAUAGCAAdTdT
U1	ACAGCATATGGCATTGCACAG	TGCAATTAAAGGTCAAAGCTGACT
U2	GTGCTTAACGTTTTTGGTCAAAGTT	GCCATCTAACAACACCCAAGT
U3	CCTGTGGATGCCACCTCTTA	AGAGGAGGCACTAAACGTGTAATAA
U4	AGACTTCCCATTGCAAGCATA	AACTCTAAGCACACCCAGCA
U5	GGCCAGGTTGTTAAAGCACT	CATCGGCCAGAACTCTTAGC
U6	GCTCTGTCACCAACACTCACT	GCTGCCTTTCTTGTGTCGTT
D1	CGAGATTCCCTTGCTGTCTCC	CCTCATATGTGGAATGGGGGAT
D2	GTGACTCACGTCAGCACCTG	ACATCGAGAGATACTCTTGTGGAC
U6-pGL	ATGGGGCCTCATTTGTTCCC	GAAGATGAACTCTGCGTGGC

D1-pGL	CAGTGTTATGCTGTTTCGCACA	AGCCAGATTGTGCAAGAGTC
Mut-D1	GTTTTCCCCAAAGGTTTGTTTCTATTTAC TTTTTCAACTTTGATC	GAAACAAACCTTTGGGGAAAACACTCCCAGA GGGAACAAAAAAAAGG
Mut-D2	TTTTCCCCAAAGGTTTCCCGTTAAAA TTAAGGTCAAAATGTCCAC	AACGGGAAACCTTTGGGGAAAAGTATTTAAGA GTTAGCATGGTGTAC
NC	ACCCTCGTTGCCAAGAATCTA	ACTACCAGCAATATAAGCAGCCA
3′UTR RBAK	TCAAAGATCCCTCAAGTGCCT	GATCTGCAATCCACCTCCAA
3′UTR MMP11	GACTGAGCCCATGTCTCCTC	AGAAAACAACTGTGTTTAATGACAA
3′UTR RBAK-mut	TGTTATGGTTACACAGAAATAATGAA GGTGTTAATTTGGC	ATTTCTGTGTAACCATAACAGGTCTTGATATTT AGAAAT
3′UTR MMP11-mut	TCCTCCGGTTACACTGTAAATGTGTGTAC AGTGTGTATAAACC	TTTACAGTGTAACCGGAGGATAGCAGTGCTGCGA AAAGGGCTT
hsa-mir-135a mimic	UAUGGCUUUUUAUUCCUAUGUGA	ACAUAGGAAUAAAAAGCCAUAUU
hsa-mir-135a inhibitor	UCACAUAGGAAUAAAAAGCCAUA	
